# Ultra-high-throughput Ca^2+^ assay in platelets to distinguish ITAM-linked and G-protein-coupled receptor activation

**DOI:** 10.1016/j.isci.2021.103718

**Published:** 2021-12-31

**Authors:** Delia I. Fernández, Isabella Provenzale, Hilaire Y.F. Cheung, Jan van Groningen, Bibian M.E. Tullemans, Alicia Veninga, Joanne L. Dunster, Saman Honarnejad, Helma van den Hurk, Marijke J.E. Kuijpers, Johan W.M. Heemskerk

**Affiliations:** 1 Department of Biochemistry, Cardiovascular Research Institute Maastricht (CARIM), Maastricht University, 6229 ER Maastricht, The Netherlands; 2 Platelet Proteomics Group, Center for Research in Molecular Medicine and Chronic Diseases (CiMUS), Universidad de Santiago de Compostela, 15782 Santiago de Compostela, Spain; 3 Institute for Cardiovascular and Metabolic Research, University of Reading, RG6 6AX Reading, UK; 4 ISASLeibniz-Institut fur Analytische Wissenschaften-ISAS-e.V., 44227 Dortmund, Germany; 5 Institute of Cardiovascular Sciences, Institute of Biomedical Research, College of Medical and Dental Sciences, University of Birmingham, Birmingham B15 2TT, UK; 6 Pivot Park Screening Centre, 5349 AB Oss, the Netherlands; 7 Thrombosis Expertise Centre, Heart and Vascular Centre, Maastricht University Medical Centre, Maastricht, the Netherlands; 8 Synapse Research Institute, Kon. Emmaplein 7, 6214 AC, Maastricht, the Netherlands

**Keywords:** Cell biology, Functional aspects of cell biology, Methodology in biological sciences

## Abstract

Antiplatelet drugs targeting G-protein-coupled receptors (GPCRs), used for the secondary prevention of arterial thrombosis, coincide with an increased bleeding risk. Targeting ITAM-linked receptors, such as the collagen receptor glycoprotein VI (GPVI), is expected to provide a better antithrombotic-hemostatic profile. Here, we developed and characterized an ultra-high-throughput (UHT) method based on intracellular [Ca^2+^]_i_ increases to differentiate GPVI and GPCR effects on platelets. In 96-, 384-, or 1,536-well formats, Calcium-6-loaded human platelets displayed a slow-prolonged or fast-transient [Ca^2+^]_i_ increase when stimulated with the GPVI agonist collagen-related peptide or with thrombin and other GPCR agonists, respectively. Semi-automated curve fitting revealed five parameters describing the Ca^2+^ responses. Verification of the UHT assay was done with a robustness compound library and clinically relevant platelet inhibitors. Taken together, these results present proof of principle of distinct receptor-type-dependent Ca^2+^ signaling curves in platelets, which allow identification of new inhibitors in a UHT way.

## Highlights

• Tools are needed to screen for the anti-thrombotic potential of small molecules
• Ultra-high-throughput assay to discriminate between GPVI and GPCR pathways
• Calcium signaling as a profiling method to follow real-time platelet activation
• Platelet calcium assays are highly sensitive to evaluate inhibitory drug effects

## Graphical abstract

**Figure:**
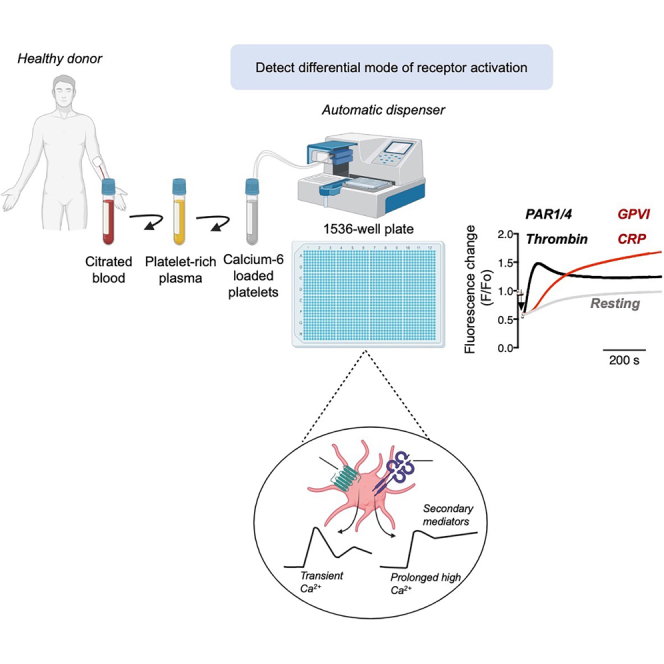

## Introduction

Antiplatelet therapy is a frontline antithrombotic strategy in the secondary prevention of arterial thrombosis (Van der Meijden and Heemskerk, 2019). All currently used oral anti-platelet drugs target G-protein-coupled receptor (GPCR) pathways, such as those evoked by the ADP receptor P2Y_12_ (clopidogrel, ticagrelor, prasugrel) and the prostanoid TP receptor (aspirin, inhibiting formation of the TP receptor's ligand thromboxane A_2_) (Michelson, 2010; Anderson and Morrow, 2017). However, these drugs, especially in dual therapy, increase the risk of bleeding in a substantial number of patients (Cattaneo, 2004; McFadyen et al., 2018). In development are antagonists of the GPCR activated by thrombin, protease-activated receptor (PAR)1 and 4, but their antithrombotic profile is still unclear (McFadyen et al., 2018; Van der Meijden and Heemskerk, 2019). This urges for a continued need to discover and develop drugs that target the role of platelets in thrombosis without compromising hemostasis (Gachet, 2015).

In addition to GPCR, also the ITAM-linked receptor (ILR) for collagen, glycoprotein (GP)VI, regulates platelet activation (Nieswandt et al., 2001). In mouse, deficiency of platelet GPVI suppresses arterial thrombosis with limited effect on hemostasis (Baaten et al., 2018; Fernandez et al., 2020). Similarly, in patients with a genetic GPVI defect only a mild bleeding phenotype is observed (Matus et al., 2013), in contrast to the major bleedings observed in patients with defective ADP receptors (Nurden and Nurden, 2015). Recent clinical trials with antibody-based or recombinant protein-based drugs to target the GPVI interaction with collagen have provided proof-of-principle evidence for the antithrombotic potential of such ILR-affecting compounds (Ungerer et al., 2011; Voors-Pette et al., 2019). However, because of the immunogenicity of antibody-based drugs, there is continued interest in searching for small molecules with effective drug-like properties (Arkin et al., 2014; Gurevich and Gurevich, 2014). To facilitate the search of large libraries of small molecule compounds for antiplatelet effects, methods in ultra-high-throughput (UHT) screening format need to be developed that can discriminate between GPCR- and ILR-induced platelet responses.

In platelets, elevated cytosolic free Ca^2+^ concentration [Ca^2+^]_i_ acts as a common second messenger in response to both GPCR and ILR agonists. Agonist-induced increases in [Ca^2+^]_i_ precede essentially all functional platelet responses, such as cytoskeletal reorganization, platelet adhesion, aggregation, secretion, and procoagulant and proinflammatory activity, and clotting (Versteeg et al., 2013; Van der Meijden and Heemskerk, 2019). A substantial amount of literature has detailed the upstream signaling and downstream effects of [Ca^2+^]_i_ rises for platelet functions after stimulation of GPCRs with thrombin and ADP (Sargeant and Sage, 1994; Heemskerk et al., 1993; Gachet, 2001; Offermanns, 2006; Covic et al., 2000), and after simulation of the ILR GPVI (Siljander et al., 2004; Varga-Szabo et al., 2009; Mammadova-Bach et al., 2019). In brief, the platelet agonists thrombin, TRAP6, ADP, and thromboxane A_2_ all stimulate platelets via Gqα and phospholipase Cβ (PLCβ) isoforms, resulting in cytosolic Ca^2+^ mobilization (via inositol triphosphate formation and intracellular Ca^2+^ release from the endoplasmic reticulum) and PKCα/β activation. The agonist CRP for ITAM-linked GPVI acts in a tyrosine-kinase dependent way, which results in activation of the PLCγ2 isoform and again causes inositol trisphosphate-mediated Ca^2+^ mobilization and PKCα/β activation. A common subsequent event is store-operated Ca^2+^ entry (Mammadova-Bach et al., 2019). Although it is understood that the GPCR-induced platelet signaling response is of shorter duration than the GPVI-induced response (Fernandez et al., 2020; Zou et al., 2021), this difference has not been examined in detail so far. In the present paper, we hypothesized that detailed analysis of the cytosolic free Ca^2+^ dynamics can help in the screening for drugs discriminating between platelet GPCR and ILR agonists.

Historically, agonist-induced [Ca^2+^]_i_ increases in platelets are measured in cuvette-based assays using the fluorescent ratio dye Fura-2, allowing one to record calibrated [Ca^2+^]_i_ responses in platelets from healthy subjects or patients (Sargeant and Sage, 1994; Heemskerk et al., 1997; Nagy et al., 2018). Single-wavelength dyes such as Fluo-4 provide reliable quantitative information on platelet Ca^2+^ responses by pseudo-ratioing analysis (Heemskerk et al., 2001). Since several years, robot instruments such as FlexStation 3 and FLIPR-Tetra are available to enable the simultaneous fluorescence analysis of multiple cell samples in 96-well plates (Bye et al., 2018). However, their application for a proper comparison of GPCR- and ILR-induced signaling is still missing.

In the present study, we developed a UHT-based method for platelet [Ca^2+^]_i_ increases using the dye Calcium-6, for operation in 384- and 1,536-well formats to establish the characteristics of Ca^2+^ signaling and to evaluate the effects of antiplatelet drugs. To achieve this, we downscaled the platelet sample size to 6 μL and used robot machines to automatically stimulate dye-loaded platelets with agonist and to read fluorescence changes in multiple wells at a time. We evaluated key GPCR agonists (thrombin, TRAP6, U46619, 2-MeS-ADP) and the ILR agonist, collagen-related peptide (CRP). After establishing time-dependent outcome parameters, we validated these using a robustness compound library and by a reference set of clinically relevant antiplatelet agents. Our results provide proof-of-principle evidence that new receptor-type-dependent antiplatelet drugs can be identified by this UHT screening.

## Results

### High-throughput assessment of [Ca^2+^]_i_ increases in CRP- and thrombin-stimulated platelets with Calcium-6

**Figure 1:**
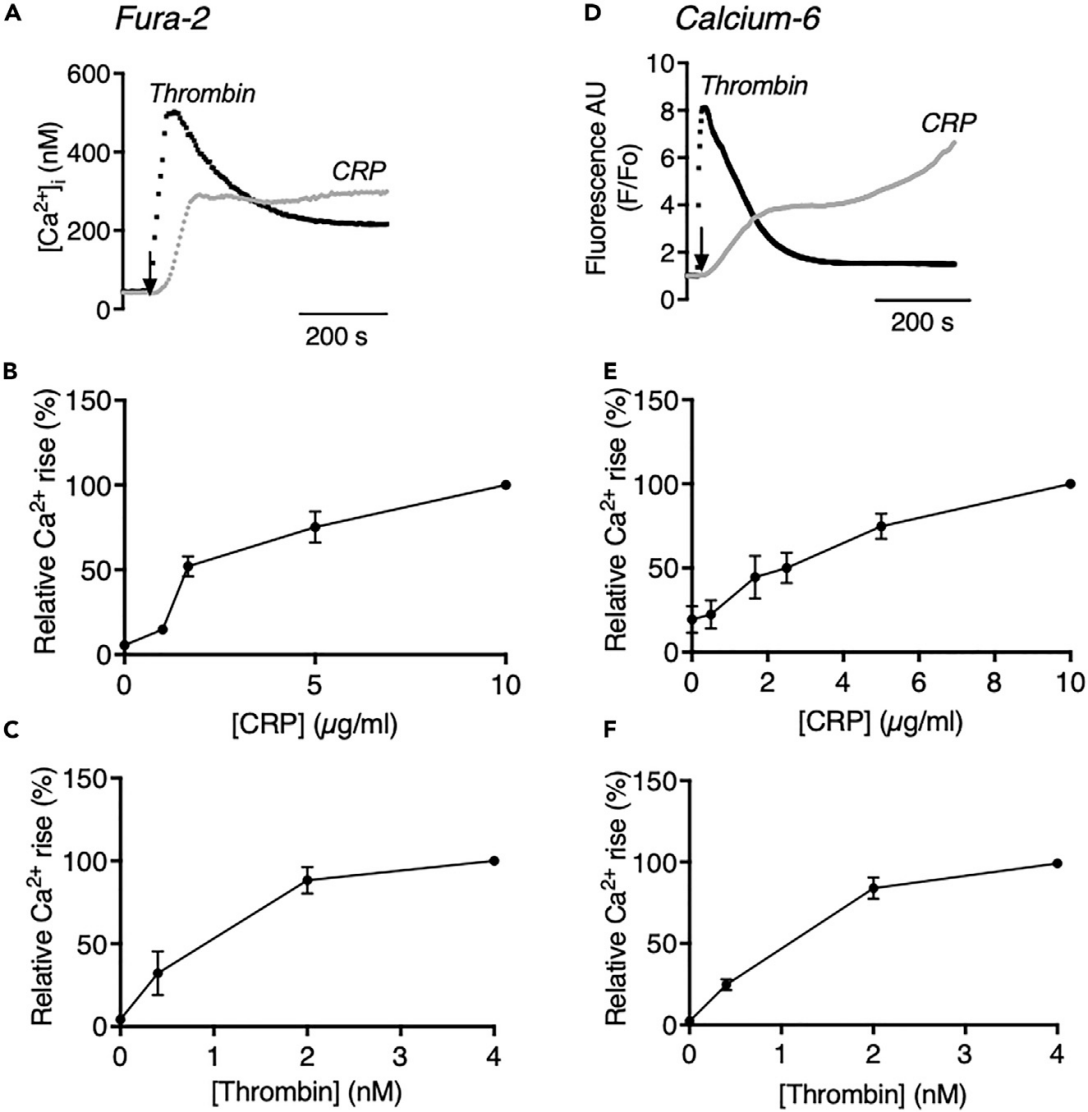
Comparison of agonist-induced [Ca^2+^]_i_ increases of platelets loaded with Fura-2 or Calcium-6 in 96-well format (A–F) Washed human platelets (200 × 10^9^ platelets/L) were loaded with Fura-2 (A–C) or Calcium-6 (D–F). Aliquots in 96-well plates were evaluated for changes in fluorescence upon stimulation with maximally effective CRP (10 μg/mL) or thrombin (4 nM) by automated pipetting in a FlexStation 3 robot. (A) Calibrated nanomolar increases in [Ca^2+^]_i_ with Fura-2 by 340/380 nm ratio fluorometry. (D) Pseudo-ratioed F/F_o_ increases with Calcium-6 obtained by single wavelength recording. Shown are representative traces. (B and E) Dose-response curves of [Ca^2+^]_i_ increase with CRP (0.5–10 μg/mL), expressed as % of maximal increase. (C and F) Dose-response curves of [Ca^2+^]_i_ increase with thrombin (0.4–4 nM), expressed as % of maximal increase. Means ± SEM, n = 3–6 donors.

For comparison with a UHT assay of agonist-induced Ca^2+^ responses, washed human platelets were first loaded with the ratiometric dye Fura-2, thus allowing measurement of calibrated nanomolar changes in [Ca^2+^]_i_ (Mahaut-Smith et al., 2000; Sage et al., 2011). These Fura-2-loaded platelets were then screened for agonist-induced responses in the 96-well plate format (200 μL of 200 × 10^9^platelets/L) using a FlexStation 3 robot, which allowed the simultaneous measurement of one column at a time. The agonists were added by high-speed injection in order to obtain reproducible and highest responses. This resulted in relatively rapid and partially transient [Ca^2+^]_i_ traces in response to the GPCR agonist thrombin, but in slower and more persistently high [Ca^2+^]_i_ traces with the ILR agonist CRP (Figures 1A–1C), which is in agreement with earlier results using dye-loaded platelets in 96-well plates (Bye et al., 2018; Jooss et al., 2019). A limitation of Fura-2 is its high sensitivity to light-absorbing compounds below 480 nm.

To overcome this limitation, we determined again in 96-well format the high-wavelength dye Calcium-6, also loaded as acetoxymethyl ester with low leakage rate after de-esterification and commonly used in high-throughput assays with other cell types (Daily et al., 2017). Platelet suspensions loaded with Calcium-6 again showed a fast and transient Ca^2+^ response upon injection with thrombin and a slow-onset prolonged biphasic Ca^2+^ response upon injection with CRP (Figures 1D–1F). Pseudo-rationing was applied to obtain F/F_0_ time curves, representing the relative increases in [Ca^2+^]_i_ (Heemskerk et al., 2001). The higher K_d_ (330 nM) of Calcium-6 for Ca^2+^ in comparison with Fura-2 (224 nM) may be responsible for the small differences in response curves between probes. The clearly distinct shapes obtained with thrombin and CRP motivated us to further explore the use of Calcium-6 as an intracellular Ca^2+^ dye.

Next, we established dose-response curves by taking the maximal calibrated or pseudo-ratioed [Ca^2+^]_i_ increases over 10 min as an output parameter. For Fura-2 and Calcium-6, EC_50_ concentrations were highly similar for both agonists, i.e., 2–3 μg/mL for CRP (Figures 1B and 1E) and 1–1.5 nM for thrombin (Figures 1C and 1F). This was confirmed by dose-response analyses based on the curve slope or the area-under-the-curve parameters (data not shown). Thus, we concluded that Calcium-6 adequately monitored the distinct Ca^2+^ responses of platelets dispersed over 96-well plates.

### Suitability of the 384-well plate format for measuring agonist-induced [Ca^2+^]_i_ increases

**Figure 2:**
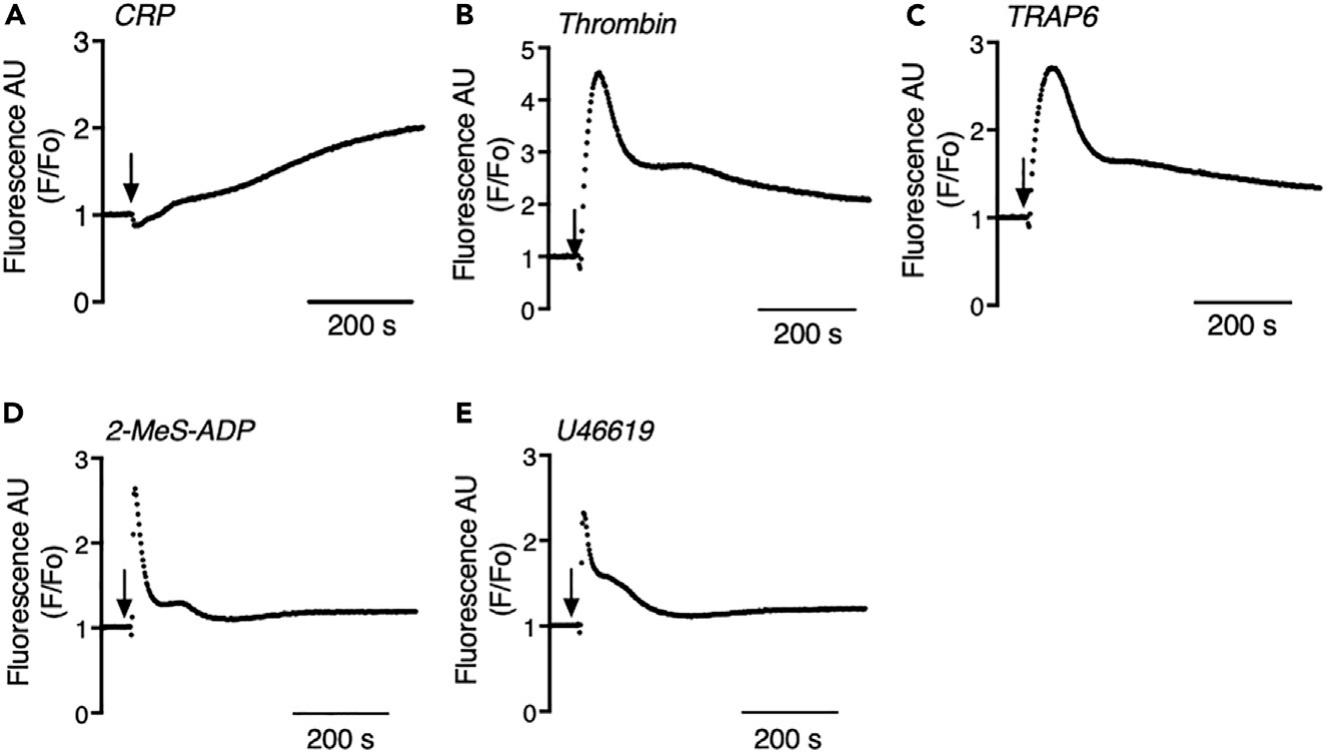
Increases in [Ca^2+^]_i_ in Calcium-6-loaded platelets by range of agonists in 384-well format (A–E) Calcium-6-loaded platelets (200 × 10^9^ platelets/L) were injection stimulated with physiologically relevant receptor agonists CRP 10 μg/mL (A), thrombin 4 nM (B), TRAP6 10 μM (C), Me-S-ADP 10 μM (D), or U46619 10 μM (E) in 384-well plates. Arrows indicate addition of agonists. Pseudo-ratioed increases in F/F_o_ were measured using a FLIPR-Tetra robot over 600 s. Representative traces of at least three experiments.

To achieve a higher throughput, we downscaled the test volume to 50 μL per well of Calcium-6-loaded platelets using 384-well plates. A camera-based FLIPR-Tetra robot was used, which allowed quantification of the fluorescence changes from all wells at the same time. The platelet concentration was kept at 200 × 10^9^/L as a compromise between sufficient fluorescent signal intensity and the availability of cells. In the 384-well format and quadruplicate wells, we compared the Ca^2+^ responses to commonly used GPCR agonists, i.e., thrombin (activating PAR1/4), TRAP6 (for PAR1), 2-MeS-ADP (for P2Y_1_ receptor), and U46619 (for TP receptor). Agonist addition again was by optimized high-speed injection with the robot. Markedly, all GPCR agonists showed faster and more transient [Ca^2+^]_i_ increases, when compared with the slower-onset and prolonged [Ca^2+^]_i_ increases obtained with CRP (Figures 2A–2E). As expected, the Ca^2+^ signals of weak agonists, 2-MeS-ADP and U46619 (Figures 2D and 2E), were lower and shorter in duration than those with more potent PAR agonists. To further characterize the differences between GPCR and ILR (CRP)-mediated Ca^2+^ responses, in the same 384-well format we investigated the effect of the Syk tyrosine kinase inhibitor PRT060318. Markedly, this compound increased the Ca^2+^ responses with all four GPCR agonists but completely annulled the Ca^2+^ response with CRP (Figure S1). Additional control experiments indicated that (1) the CRP-induced Ca^2+^ signal was completely dependent on tyrosine kinase signaling (Figure S2A), (2) the thrombin-induced Ca^2+^ signal was fully blocked by treatment with the catalytic site thrombin inhibitor, PPACK (Figure S2B), and (3) the addition of 1 mM extracellular CaCl_2_ to the dye-loaded platelets in wells was sufficient to reach a plateau level of intracellular Ca^2+^ signals with both CRP and thrombin (Figures S2C and S2D). Dosing effects of the two agonists were similar to those in 96-well plates (see Figure 1).

### UTH assay miniaturization to 1,536-well plates and quality analysis

**Figure 3:**
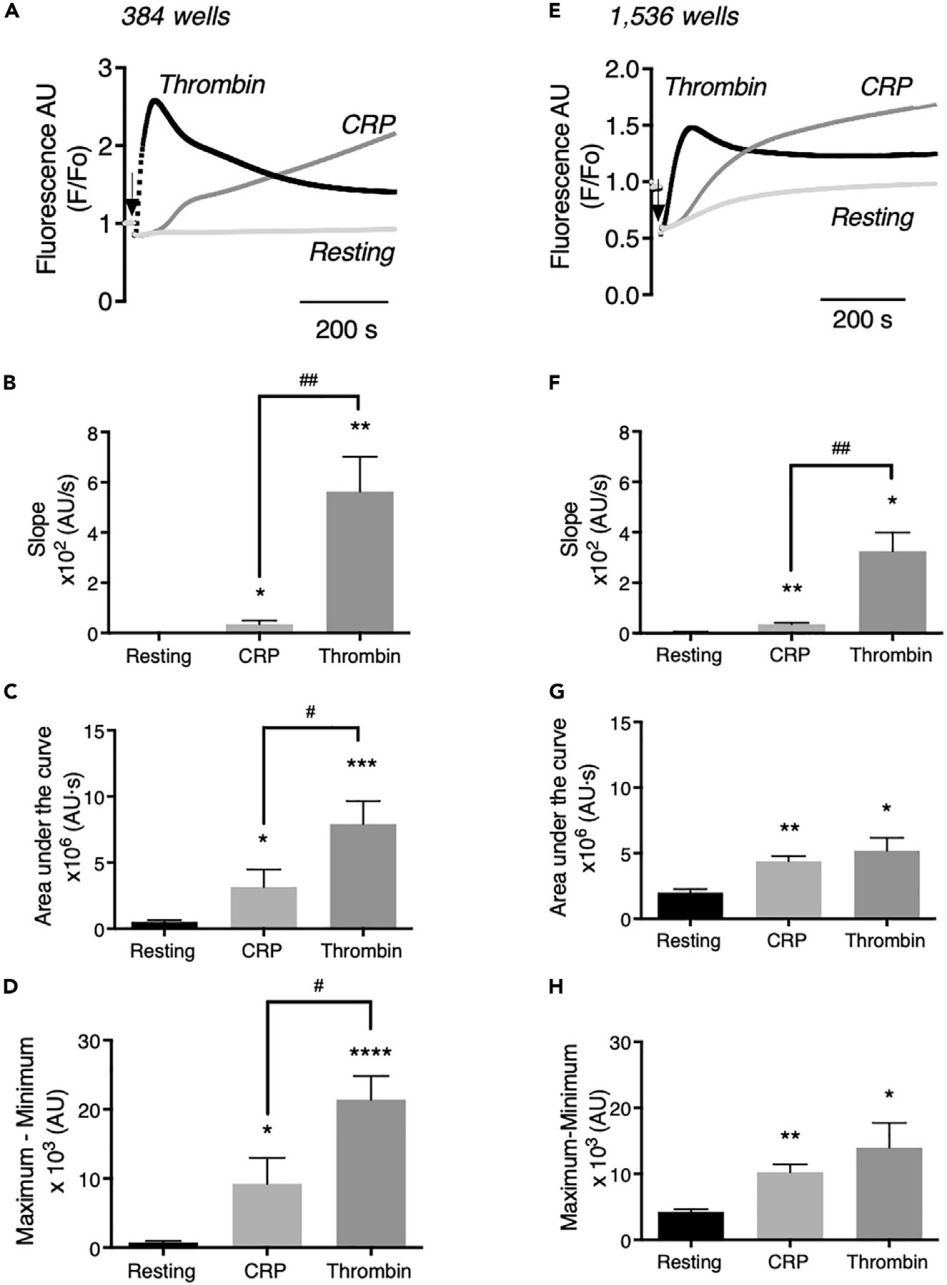
Comparative thrombin- and CRP-induced [Ca^2+^]_i_ increases of platelets in 384- and 1,536-well formats Using 384- or 1,526-well plates, Calcium-6-loaded platelets were stimulated with CRP (10 μg/mL, final concentration), thrombin (4 nM, final concentration), or vehicle medium (resting). Agonist injection volume and rate were optimized per well-plate format to obtain highest fluorescence increases with the FLIPR-Tetra machine. Time-dependent traces per well were constructed of pseudo-ratio fluorescence (F/F_o_), indicative of changes in [Ca^2+^]_i_. (A–D) Results from 384-well plates with 50 μL platelets (200 × 10^9^/L) and 5 μL agonist solution injected. Data are means ± SEM (n = 6). (E-H) Results from 1,536-well plates with 6 μL platelets (400 × 10^9^/L) and 2 μL agonist solution injected. Means ± SEM (n = 3). (A and E) Representative [Ca^2+^]_i_ traces of resting and CRP- or thrombin-stimulated platelets. (B and F) Measured slopes of pseudo-ratioed increases in fluorescence. (C and G) Maximum–minimum increases with CRP (over 600 s) or thrombin (peak level). Minimal fluorescence levels were determined after injection to exclude dilution effects. (D and H) Area under the curve of response over 600 s. One-tailed Student's t test, ∗p < 0.05, ∗∗p < 0.01, ∗∗∗p < 0.001, ∗∗∗∗p < 0.0001 versus resting; ^#^p < 0.05, ^##^p < 0.01 CRP versus thrombin.

Aiming to further downscale the assay, we moved to the 1,536-well format, which was possible by using the FLIPR-Tetra machine. Per well, 6 μL of Calcium-6-loaded platelets (concentration doubled to 400 × 10^9^/L) was used, and a minimal volume of 2 μL of agonist solution was injection pipetted for achieving maximal responses. For analysis again pseudo-ratioing was applied, which in this case resulted in a sudden drop in fluorescence intensity due to the 33% dilution with agonist. A direct comparison of the overall assays' performances in 384- and 1,536- well formats is shown in Figure 3. Notably, in the 1,536-well format, the different fluorescence kinetics in response to thrombin (fast transient) and CRP (slow persistent) were well maintained. The use of relatively high concentrations of platelets showed a small gradual increase in fluorescence over 10 min in resting platelets, which was ascribed to dye leakage. Single-donor comparison of Calcium-6-loaded platelet responses in 384- and 1,536- well plates showed the same typical shape differences in responses to thrombin and CRP (Figure S3). For the same batch of platelets, it was also confirmed by flow cytometry that CRP and thrombin addition caused functional responses such as integrin αIIbβ3 activation (PAC1 mAb binding) and granular secretion (P-selectin expression) (Figure S4).

For detailed analysis of the pseudo-ratioed F/F_o_ [Ca^2+^]_i_ traces, we examined three parameters: (1) maximal curve slope after agonist addition, (2) maximal fluorescence increase over time (maximum - minimum), and (3) area under the curve (AUC), the latter as an approximation of the net integrated [Ca^2+^]_i_ increase over 10 min. Quantification showed that curve slopes were 16- and 9-fold higher with thrombin than with CRP, when measured in 384- (Figures 3A and 3B) or in 1,536-well plates (Figures 3E and 3F), respectively. The ratio of AUCs for thrombin versus CRP was 2.3 and 1.2 for 384- and 1,536-well formats (Figures 3C and 3G), and the maximal [Ca^2+^]_i_ increases with thrombin were 2.5- and 1.4-fold higher than with CRP in 384- or 1,536-well plates, respectively (Figures 3D and 3H).

**Figure 4:**
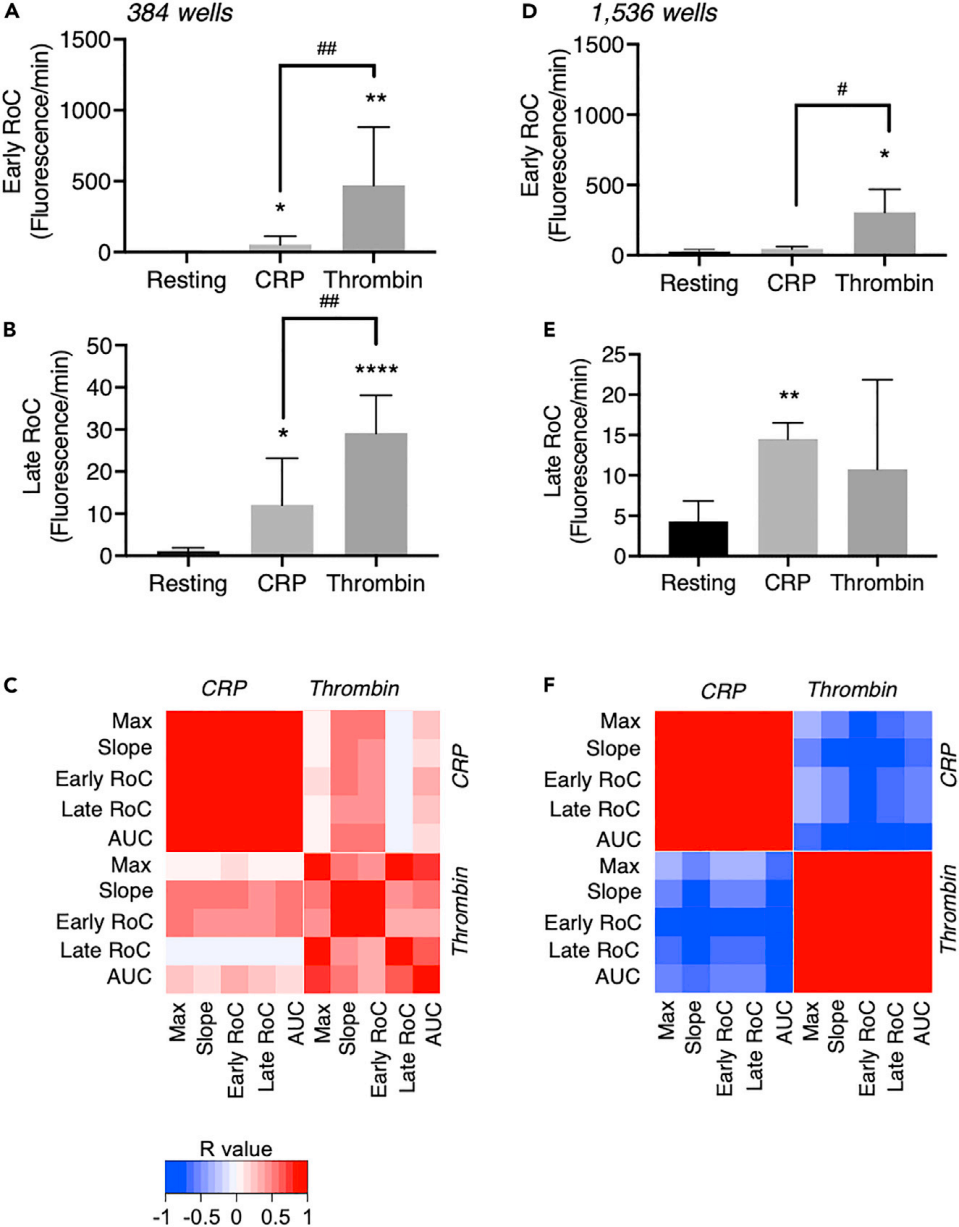
Comparative analysis of [Ca^2+^]_i_ curve parameters of thrombin- and CRP-induced platelet responses in 384- and 1,536-well formats (A–F) Calcium-6-loaded platelets in 384-well (A–C) or 1,536-well (D–F) plates were stimulated with CRP or thrombin or left unstimulated, as in Figure 3. Fluorescence changes measured for 600 s were regressed to smoothed curves for the calculation of early (A and D) and late (B and E) rates of change (RoC) of the [Ca^2+^]_i_ increases per agonist. (C and F) Pearson correlation analyses were performed for all curve parameters used for Ca^2+^ response quantification: maximum - minimum rise (Max), slope of increase (slope), early and late RoC, and area under the curve (AUC). (C and F). Shown are heatmapped Pearson correlation matrices of R values of the various curve parameters, with blue/red colors indicating negative or positive correlations for 384- and 1,536-well plates, respectively. Means ± SEM (n = 3–6). Non-parametric Mann-Whitney test (A and D) or one tailed Student's t test (B and E), ∗p < 0.05, ∗∗p < 0.005, ∗∗∗∗p < 0.0001 versus resting. (A–E) One-tailed Student's t test, ^#^p < 0.05, ^##^p < 0.01 CRP versus thrombin.

In order to further dissect the different shapes of the curves, we fitted these by obtaining smoothed, average-weighted curves. We then determined the rate of change (RoC) after agonist addition. We furthermore separated the biphasic curves into two parts, resulting in an early RoC (over 50–200 s) and a late RoC (over 200–450 s). The late RoC was considered as a best estimate of the later second-phase [Ca^2+^]_i_ response. When comparing the 384- and 1,536-well formats, the early RoC was invariably higher with thrombin than with CRP, in agreement with the observed fast onset (Figures 4A and 4D). The late RoC was similar in size with thrombin and CRP in either well-plate format (Figures 4B and 4E).

Comparing the effects of CRP and thrombin, we observed that slope and early RoC significantly differed in 384- and in 1,536-well plates (p < 0.01), while the other parameters were significantly different for CRP and thrombin only in 384-well plates (p < 0.05). Accordingly, slope was the parameter that most consistently discriminated between the two agonists. A regression analysis of all curve parameters obtained with platelets from all investigated donors indicated positive correlations with low p values (p < 0.05) for maximal signal, slope, areas under the curve, and early/late RoC in case of CRP or thrombin, regardless of the type of well plate (Figures 4C and 4F). However, this correlation was absent between both agonists in 384-well plates. In 1,536-well plates, negative correlations were observed between the agonists, because the agonist-specific effects of early and late increases operating at different parts of the curves were more prominent in the 1,536-well format. This confirmed the different trace characteristics between both agonists.

To evaluate the overall measurement performance per type of well plate, we calculated the Z′ factor, as a combined measure of the signal dynamic range and the intra-test variation (Zhang et al., 1999). In 384 wells, the Z′ factors for the maximal signal were within the range of excellence (1 > Z' ≥ 0.5), i.e., 0.63 ± 0.03 for CRP and 0.71 ± 0.06 for thrombin (means ± SEM, n = 6). For the Calcium-6-loaded platelets in 1,536 wells, Z′ factors were slightly lower, i.e., 0.39 ± 0.13 for CRP and 0.37 ± 0.14 for thrombin (means ± SEM, n = 3). For the 1,536-well format, this pointed to a somewhat higher, but still acceptable, curve variation between wells.

### Robustness assessment of UHT measurement

The success of a UTH campaign relies on effective hit-triaging evaluation. It is important to check for a method’s liabilities to detect undesired properties of chemical compounds with assay interfering properties (Honarnejad et al., 2021). The Pivot Park Screening Centre has created a proprietary robustness set compound collection for evaluation of a wide variety of UHT assays (Honarnejad et al., 2021). The collection comprises 263 DMSO-soluble compounds with well-documented interference in different types of tests. The compounds were classified as aggregator, metal ion chelator, fluorescent, luciferase quenching, chemically reactive, metals, colored or visible light absorbing, redox active, and salt compounds. The set further contained clean compounds, which are chemically diverse lead-like compounds without known assay interfering properties. In order to evaluate the liability of the UHT platelet Ca^2+^ assay, we determined the effects of these 263 compounds in 4 quadruplicate wells per compound, spread over the 1,536-well plate.

**Figure 5:**
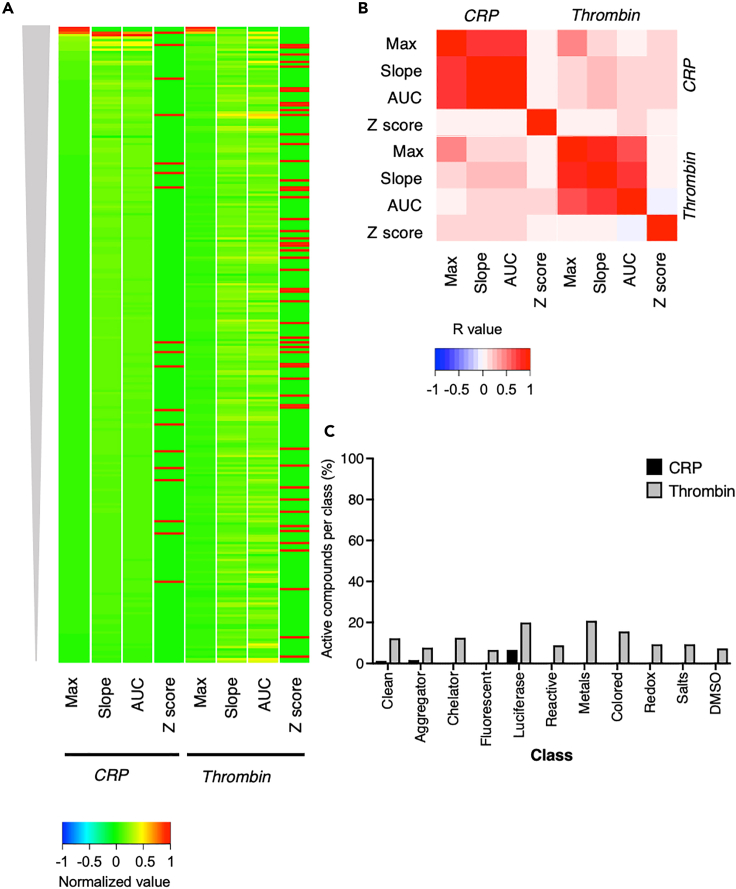
Robustness assessment of CRP- and thrombin-induced measurements of Calcium-6-loaded platelets in 1,536-well plates Increases in [Ca^2+^]_i_ of Calcium-6-loaded platelet concentrates in response to CRP (10 μg/mL) or thrombin (4 nM) were measured in 1,536-well plates. Multiplicate wells (n = 4) were preincubated with one of 263 compounds from a robustness set compound library (all 10 μM). Compounds were classified according to their potential assay interference: clean compounds (non-interfering), aggregating, metal ion chelating, fluorescent, luciferase quenching, chemically reactive, metal, colored or visible light absorbing, redox active, salt compounds, and DMSO controls. (A) Univariate scaled heatmap (−1 to 1) of mean interference of all 263 compounds in [Ca^2+^]_i_ increases induced by CRP or thrombin. Effects are represented on maximal increase (Max), initial slope (slope), area under the curve (AUC), and *Z* score. Compounds were clustered according to decreasing size effects on maximal increase. (B) Spearman correlation analysis of the four curve parameters for CRP and thrombin. Color bar represents calculated R values. (C) Percentages of active, assay-interfering compounds per class (*Z* score >4 or < -4), calculated as means of three parameters for CRP- (black) and thrombin- (gray) stimulated platelets. For further details, see Figure S1.

For this purpose, platelets from concentrates were loaded with Calcium-6 for CRP- and thrombin-induced [Ca^2+^]_i_ increase measurements. Effects of the agonists on functional platelet responses were confirmed by flow cytometry (not shown). For all the 263 investigated compounds, effect values in quadruplicate were obtained of the three curve parameters (maximal increase, initial slope, area under the curve) upon stimulation with thrombin or CRP. For mean effects of these three parameters, the *Z* score was calculated (number of standard deviations) in order to identify actively interfering compounds. Presentation of the data as a univariate scaled heatmap showed that the majority of compounds were without relevant effect, i.e., with ǀZǀ <4 (Figure 5A). Regression analysis using all compound-modified curves showed again high positive correlations between the various parameters per agonist, but not between agonists (Figure 5B).

Evaluating the Z scores for all 263 compounds per class indicated some differences between CRP- and thrombin-induced [Ca^2+^]_i_ curves. For the majority of compound classes, interference in terms of Z scores was lower in CRP- than in thrombin-stimulated conditions (Figure S5). Restricting the set to active compounds (compounds with ǀZǀ >4) indicated that, for CRP, some luciferase compounds were assay interfering (7% active compounds) as well as an aggregator compound (2%). For thrombin, on average 12% of active compounds per class were interfering (Figure 5C). Classes with the highest interfering rates were chelators (12.5%), luciferase quenching (20%), metals (21%), and colored (16%). Furthermore, the data pointed to a lower predicted hit rate for CRP (1%), based on the percentage of actives in the clean class, than for thrombin (12%). Overall, the CRP-induced Ca^2+^ signal was less liable, as only some aggregator and luciferase compounds interfered with the assay in comparison with the thrombin assay with more compound classes interfering. The protein identity of thrombin as a large 37.4-kDa protease, sensitive to catalytic or regulatory site inactivation by chemical compounds, can explain its higher sensitivity to inhibition. Overall, this screening analysis pointed to an overall high liability of the Calcium-6 platelet UHT measurement, taking into account the intrinsic method's limitations: (1) interference of compounds with high absorbance at >480 nm (excitation wavelength of Calcium-6) and (2) compounds affecting the agonist activity. Accordingly, using an appropriate hit triage cascade, an UHT-based screening procedure for novel antiplatelet drugs has now become possible.

### Effects of specific antiplatelet drugs in UHT assay

To further confirm the usefulness and strength of the 384- and 1,536-well plate measurements, we compared the effects of established antiplatelet drugs with proven antiaggregatory effects on platelets in clinically relevant conditions (Table 1). The examined drugs were next to the tyrosine kinase Syk inhibitor PRT060318, the cyclooxygenase/thromboxane synthase inhibitor indomethacin (equivalent to aspirin), and the reversibly blocking P2Y_12_ receptor antagonist ticagrelor. Indomethacin and ticagrelor interfere with platelet activation responses via GPCRs and effectively block the effects on platelets of autocrine produced thromboxane A_2_ and ADP via P2Y_12_ signaling, respectively. The drugs were applied at maximally effective doses (Gilio et al., 2009).

**Table 1:** Overview of clinically relevant antiplatelet drugs tested

Compound	Target	Receptor pathways	Effect on platelet aggregation	(Pre)clinical outcome in mouse	References
PRT060318	Selective inhibitor of Syk tyrosine kinase	GPVI/FcRγ, CLEC2	Collagen, CRP ↓ ADP, thrombin =	In mouse, reduced arterial thrombosis with low bleeding	Jooss et al. (2019), Andre et al. (2011) and Liu and Mamorska-Dyga (2017)
Indomethacin	Reversible cyclooxygenase inhibitor	Arachidonic acid (AA)	Collagen, AA ↓ ADP, thrombin = /↓	Non-steroidal anti-inflammatory drug, acting on platelets as aspirin. Moderate risk of bleeding	Bourn and Cekanova (2018), Falcinelli et al. (2019) and Perrone et al. (2010)
Ticagrelor	Reversible P2Y_12_ receptor antagonist	P2Y_12_	ADP ↓ Collagen, thrombin = /↓	Reduced ischemic events after percutaneous coronary intervention. Moderate risk of bleeding	Gresele et al. (2017), Scavone et al. (2016) and Nergiz-Unal et al. (2010)

Information obtained from (pre)clinical studies and mouse *in vivo* thrombosis assays.

**Figure 6:**
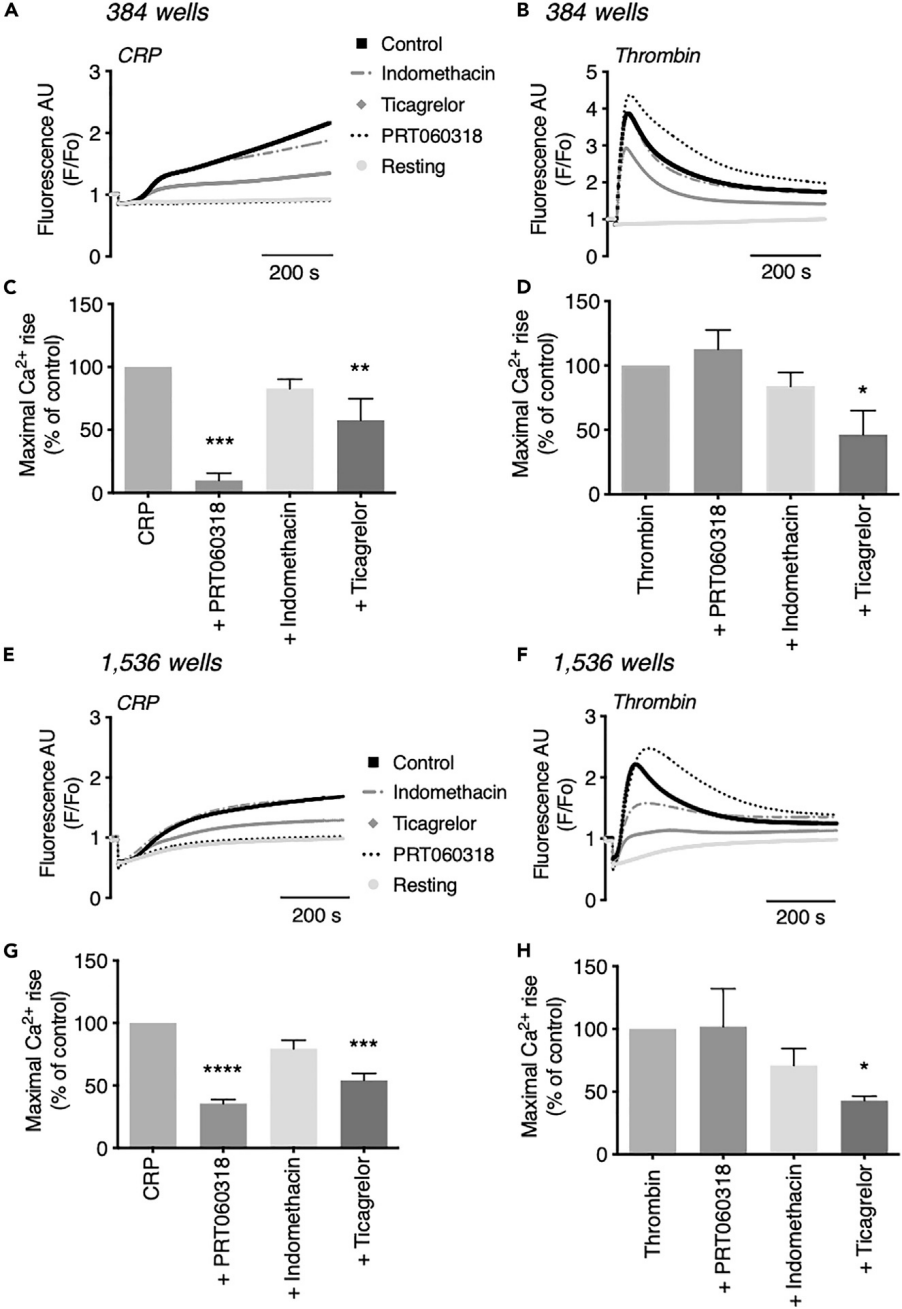
Drug-dependent inhibitory profiles on platelet Ca^2+^ responses in 384- and 1,536-well formats Calcium-6-loaded platelets were pretreated for 10 min with indicated compounds or vehicle (control) and injected with CRP (10 μg/mL) or thrombin (4 nM) or remained unstimulated (resting) using a FLIPR-Tetra robot and 384- or 1,536-well plates, as described for Figure 3. Changes in pseudo-ratio fluorescence (F/F_o_) per well were determined for 600 s. (A–D) Platelet responses in 384-well plates; means ± SEM (n = 6). (E–H) Platelet responses in 1,536-well plates; means ± SEM (n = 3). For 384-well (A and B) and 1,536-well (E and F) formats are given representative [Ca^2+^]_i_ traces of resting (gray), CRP- or thrombin-stimulated platelets. Drugs used were (final concentrations): PRT060318 (5 μM, black dotted), indomethacin (10 μM, gray dotted) or ticagrelor (10 μM, gray diamonds). Drug effects on maximum [Ca^2+^]_i_ increases in 384 wells (C and D) or 1,536 wells (G and H); values are expressed as percentages relative to corresponding control. One-way ANOVA, ∗p < 0.05, ∗∗p < 0.005, ∗∗∗p < 0.001, ∗∗∗∗p < 0.0001 versus agonist.

Comparing the drug effects on CRP- and thrombin-induced increases in [Ca^2+^]_i_ in the 384- (Figures 6A–6D) and 1,536-well plate (Figures 6E–6H) formats led to interesting results. Representative Ca^2+^ traces showed different inhibitory effects in response to CRP or thrombin, regardless of the type of well plate (Figures 6A and 6E). With CRP, the inhibition increased in the order of indomethacin < ticagrelor < PRT060318, whereas with thrombin only ticagrelor was inhibitory (Figures 6B and 6F). Systematic evaluation of the Ca^2+^ curve parameters underlined the selective inhibitory effect of PRT060318 with CRP, as described above. Indomethacin did not significantly affect the CRP- and thrombin-induced maximal Ca^2+^ signals (Figure 6). On the other hand, antagonism of P2Y_12_ by ticagrelor suppressed these signals with either agonist by 40%–60%. A control experiment showed that, with Calcium-6-loaded platelets from one donor, essentially the same effects in 384-well and 1,536-well plate formats were obtained for PRT060318, indomethacin, and ticagrelor regarding the various Ca^2+^ curve parameters (Figures S3A–S3H).

**Figure 7:**
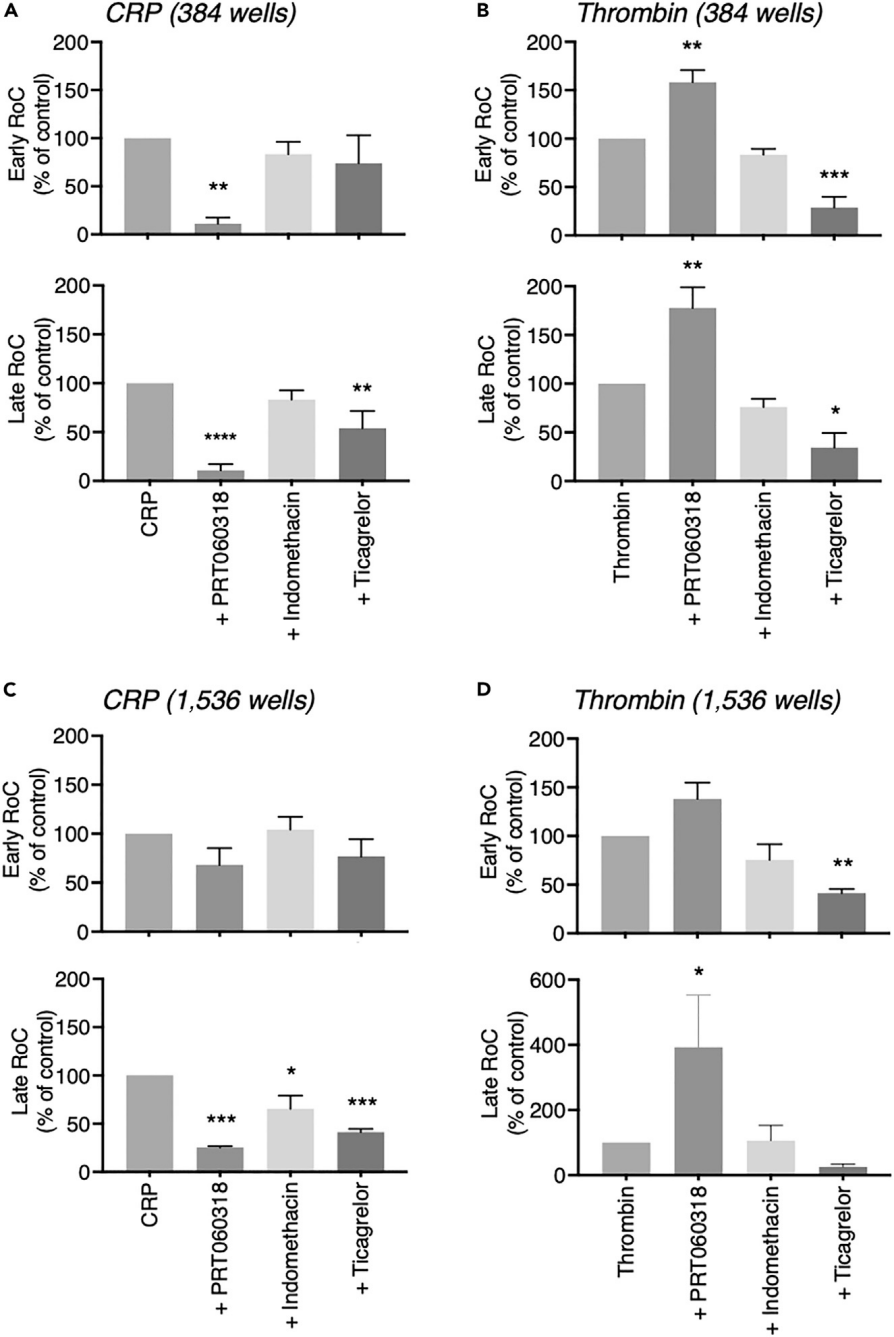
Drug-dependent effects on early and late slopes of platelet Ca^2+^ responses in 384- and 1,536-well formats Calcium-6-loaded platelets were pretreated for 10 min with PRT0606318, indomethacin, or ticagrelor and activated with CRP or thrombin in 384- wells or 1,536-well plates, as described for Figure 6. Pseudo-ratioed fluorescence traces over 600 s were regressed to smoothed curves for the calculation of early and late rates of change (RoC). (A–D) Shown are data of early RoC (upper panels) and late RoC (lower panels) for platelets stimulated with CRP (A and C) or thrombin (B and D) in 384- or 1,536-well formats. Values are expressed as percentages relative to controls. Means ± SEM (n = 3). One-way ANOVA, ∗p < 0.05, ∗∗p < 0.005, ∗∗∗p < 0.001, ∗∗∗∗p < 0.0001 versus agonist.

In order to distinguish between early and late contribution of the thromboxane A_2_ and ADP/P2Y_12_ pathways to the [Ca^2+^]_i_ curves, we again determined the early and late “slopes” as RoC per agonist and well-plate format (Figure 7). The expected strong inhibition by PRT060318 of CRP-stimulated platelets was observed in both the early and late RoCs (Figures 7A and 7C). In contrast, PRT060318 enhanced both the early and late RoCs of thrombin-stimulated platelets (Figures 7B and 7D). Analyzing the smoothed curves, indomethacin did not notably change the early or late RoCs. In contrast, ticagrelor lowered the late RoC in response to CRP and the early RoC in response to thrombin. This pointed to a more prominent role of autocrine produced ADP (acting via P2Y_12_ receptors) than of released thromboxane A_2_ (inhibited with indomethacin) under the assay conditions. To control for the effects of indomethacin on functional platelet responses, we measured in parallel experiments platelet aggregate formation using washed platelets or PRP under stirred conditions. As shown in Figure S6, indomethacin moderately lowered the CRP-induced platelet aggregation of washed platelets but not of PRP, whereas it did not alter the thrombin-induced aggregation. This pointed to a small contribution of the thromboxane pathway in suspensions of stirred, washed platelets. Altogether, these data supported the suitability of the UHT assays with Calcium-6-loaded platelets in 384- or 1,536-well formats for resolving agonist-dependent inhibitory effects of antiplatelet compounds.

## Discussion

In this paper, we describe a UHT assay for platelet signaling responses, operating as 96-, 384-, or 1,536-well plate formats, based on the different profiles of [Ca^2+^]_i_ increases as evoked by GPCR or ILR agonists. In 96- and 384-well plates, we detected comparable, short-term [Ca^2+^]_i_ transients induced by a range of physiologically relevant GPCR agonists: the PAR1/4 agonist thrombin, the PAR1 agonist TRAP6, the TP thromboxane receptor agonist U46619, and the P2Y_1_ receptor agonist 2-MeS-ADP. With all these agonists, the [Ca^2+^]_i_ increases were not suppressed but slightly increased by the Syk tyrosine kinase inhibitor PRT060318. Because of this response similarity, we chose to perform further UHT assay development with the GPCR agonist thrombin only. On the other hand, the commonly used ILR agonist CRP for GPVI evoked a more prolonged Ca^2+^ signal that was completely blocked by PRT060318. The ILR C-type lectin-like receptor 2 (CLEC2) was not investigated, since the known, physiological ligands such a podoplanin are no more than weak platelet agonists.

As described in the introduction, receptor-induced [Ca^2+^]_i_ increases comprise an essential signaling step, preceding almost all functional platelet responses (Fernandez et al., 2020). We developed a semi-automated way of curve profiling to separate early and late RoC values, which allowed us to better characterize and quantify the fast and transient [Ca^2+^]_i_ increases in response to GPCR and PLCβ stimulation with thrombin and the slow-persistent increases by ILR and PLCγ2 stimulation with CRP. The fast kinetics of the Ca^2+^ signal with thrombin, a ligand formed upon coagulation, is in agreement with earlier single-cuvette studies of stirred platelet suspensions (Heemskerk et al., 1997; Covic et al., 2000; Shapiro et al., 2000). The more delayed Ca^2+^ signal with CRP is compatible with the slow-onset Ca^2+^ traces of single platelets contacting the GPVI agonist collagen (Van Kruchten et al., 2012). In the present experiments, carried out at a plateaued level of extracellular CaCl_2_, we did not discriminate between separate components of the [Ca^2+^]_i_ increases, i.e., Ca^2+^ mobilization from intracellular stores and Ca^2+^ entry modes (Mahaut-Smith, 2012; Van Kruchten et al., 2012).

The conventional high throughput method in 96-well plates, as earlier described (Bye et al., 2018), still needed large sample volumes per well (200 μL, 200 × 10^9^ platelets/L). However, the 384-well measurements could be carried out at 50 μL per well (200 × 10^9^/L), whereas the 1,536-well assay only required 6 μL per well (400 × 10^9^/L). In the 384-well format, agonist-induced traces appeared to be highly reproducible, as apparent from the excellent, high Z′ factors. In the 1,536-well format, Z′ factors were slightly lower but within the acceptable range (Iversen et al., 2004). The latter was a consequence of the small volumes of platelet suspension (6 μL) and agonist (2 μL) present per well and the known inter-well variability in automated pipetting and in cell concentrations (Martis et al., 2011). In general, it is considered that the UHT-well formats enable a higher capability of testing with reduction of costs and time (Bhambure et al., 2011; Mayr and Fuerst, 2008).

Suitability of the developed UTH 1,536-well assay for screening purposes was further assessed using an in-home developed robustness compound collection. We found that the CRP-induced [Ca^2+^]_i_ traces were essentially insensitive to the set of possible assay-interfering compounds, whereas the thrombin-induced responses showed a limited but still acceptable compound interference. A likely explanation for this difference is that CRP consists of a cross-linked triple-helical peptide with a high molecular stability (Smethurst et al., 2007). On the other hand, thrombin as a large 37.4-kDa protease is by default more sensitive to catalytic or regulatory site inactivation by chemical compounds. The fraction of active, assay-interfering compounds per class of 12% is still lower than that seen in other UHT tests (Honarnejad et al., 2021). In other words, in comparison with other cell systems, the platelet [Ca^2+^]_i_ assay in the 1,536-well format does not appear to be inferior.

Application of the 384- and 1,536-well plate measurements with relevant anti-platelet inhibitors, i.e., indomethacin, ticagrelor, and PRT060318, provided detailed insight into the time-dependent contribution of autocrine-dependent signaling routes after CRP or thrombin activation. The ADP/P2Y_12_ antagonist ticagrelor suppressed only the late RoC with CRP and both the early and late RoCs with thrombin. This pointed to an unknown high contribution of the ADP/P2Y_12_ enforcement pathway in the early stage of thrombin-induced signal generation. These assay results, in agreement with platelet functional measurements, demonstrated that platelet assays based on receptor-type-specific [Ca^2+^]_i_ increases are highly sensitive to evaluate the inhibitory effects of compounds in each phase of the [Ca^2+^]_i_ curve in an agonist-dependent way.

In future, the present UHT assays in 384- or 1,536-well plate formats can be employed for large small molecule screening approaches. The 1,536-well format is the gold standard in the academic drug discovery community and the pharmaceutical industry so as to reduce costs in reagents, consumables, and biological materials and make it time efficient owing to the high number of samples (Coussens et al., 2018). So far, similar Ca^2+^-based small molecule screening has only been performed with cultured transfected cells to find novel PAR4 antagonists and as secondary assay for PAR1 antagonists (Wong et al., 2017; Dowal et al., 2011). Other UHT Ca^2+^ dye assays have been used with cardiomyocytes and stem induced pluripotent cells, among one developed by Pivot Park Screening Centre (Daily et al., 2017; Famili et al., 2019). For platelets as primary human cells, this has not yet been achieved at the level as described in our article. Of note, the useful 96-well plate Optimul assay earlier developed for measuring of platelet aggregation (Lordkipanidzé et al., 2014) uses platelet-rich plasma, has not been downscaled to 6 μL, and acts as an integral end-function test of platelet responses rather than a direct signaling test. Overall, advantages of the present UTH test are the application of receptor-dependent signaling responses in platelets, as easily accessible primary human differentiated cells; the use of a longer-wavelength Ca^2+^ probe; the absence of blood plasma; and the ability to discriminate between vital cell activation and necrosis or toxicity (resulting in unphysiologically high Ca^2+^ levels). With small adaptations, the current UHT Ca^2+^ signaling method can also be applied to cultured human cell lines and differentiated iPSCs.

In general, UHT screening assays, such as the one described here, render many positive hits, which may result not only in lead compounds but also in false-positive leads due to each particular assay (Hoelder et al., 2012; Choi and Choi, 2017). After initial compound screening, the further validation of hit antagonists involves a long process by applying several methodologies. In research to platelet GPCRs or ILR signaling inhibitors with antithrombotic potential, conventional further assays in hit-to-lead identification are light transmission aggregometry and whole-blood microfluidics assays (Provenzale et al., 2019). Bioavailability and toxicity assays as well as medicinal chemical improvements also need to be done before continuing the drug development process (Choi and Choi, 2017).

### Limitations of the study

In this study, we developed a UHT fluorescence method to measure agonist-induced [Ca^2+^]_i_ increases in platelets in 384- and 1,536-well plate formats. Herein, we were able to distinguish between GPVI- and PAR1/4-induced Ca^2+^ signaling and characterize inhibitory effects of compounds in early and late phases of the [Ca^2+^]_i_ curves. Limitations of the study are the focus on only one ILR (CRP) and GPCR (thrombin) platelet agonist in both UHT formats, absence of discrimination between individual receptor isoforms (PAR1 and 4 for thrombin), and absence of discrimination between intracellular Ca^2+^ mobilization and Ca^2+^ entry. Another limitation of such UHT assays is the absence of continuous stirring, resulting in slightly delayed responses, although mixing of platelet suspensions with agonists was optimized by high-speed injection of relatively high volumes (10%–33%) of agonist solution. On the other hand, the absence of stirring suppressed the system heterogeneity, i.e., prevented the formation of large platelet aggregates (with CRP) or fibrin clots (with thrombin). Prolonged, biphasic Ca^2+^ curves of GPVI-stimulated platelets and aberrations in these with patients carrying genetic mutations in *ORAI1* or *STIM1* were previously also obtained in stirred, single-cuvette measurements (Nagy et al., 2018). For the present paper, experimental choices were made to evaluate the full Ca^2+^ signals in response to CRP, as a widely used collagen-like GPVI agonist, and thrombin as a physiological, coagulation-dependent agonist on platelets.

## STAR★Methods

### Key resources table

**Table:** 

REAGENT or RESOURCE	SOURCE	IDENTIFIER
**Chemicals, peptides, and recombinant proteins**		

Cross-linked collagen-related peptide, CRP-XL (CRP)	CambCol Laboratories (Cambridge, UK)	Cat# CRP-XL
Fura-2-AM	Invitrogen (Carlsbad, CA, USA)	Cat# F1221
PRT060318	Bio-Connect (Huissen, The Netherlands)	Cat#P838895_5mg
Indomethacin	Sigma-Aldrich (Zwijndrecht, The Netherlands)	Cat# I7378
Human α-thrombin	Sigma-Aldrich (Zwijndrecht, The Netherlands)	Cat#T6884
Ticagrelor	Kindly provided by AstraZeneca R&D (Mölndal, Sweden)	N.a.
D-Phe-Pro-Arg chloromethyl ketone (PPACK)	Santa Cruz Biotechnology (Santa Cruz, TX, USA)	sc-201291
U46619	Cayman Chemicals (Ann Arbor, MI, USA)	Cat# D8174
2-MeS-ADP	Santa Cruz Biotechnology (Dallas, TX, USA)	Cat# sc-203464
DMSO	Sigma-Aldrich (Saint Louis, MO, USA)	Cat#D8418
Triton-X-100	Sigma-Aldrich (Saint Louis, MO, USA)	Cat#11332481001
CaCl_2_	Sigma-Aldrich (Saint Louis, MO, USA)	Cat# 223506
Alexa Fluor-647 anti-CD62P mAb	Biolegend (San Diego, California, United States)	Cat# 304918; RRID: AB_2185110
FITC-PAC1 mAb	BD Bioscience (Franklin Lakes, New Jersey, United States)	Cat# 340507; RRID: AB_2230769

**Critical commercial assays**		

FLIPR Calcium-6 Assay Kit	Molecular Devices (San Jose, CA, USA)	Cat# R8190

**Software and algorithms**		

GraphPad Prism 8	GraphPad Prism 8 (San Diego, CA, USA)	https://www.graphpad.com
R software version 3.2.5	https://www.R-project.org/	https://www.r-project.org

### Resource availability

#### Lead contact

Further information and request for resources and reagents should be directed to and will be fulfilled by the lead contact, Delia I. Fernández (d.fernandezdelafuent@maastrichtuniversity.nl).

#### Materials availability

This study did not generate new unique reagents.

### Experimental model and subject details

#### Human subjects

Blood was obtained by venipuncture from healthy male and female volunteers who had not received anti-platelet medication for at least two weeks, after full informed consent according to the Helsinki declaration. The study was approved by the Medical Ethics Committee of Maastricht University. According to the approval, blood donor age and sex were not recorded. Blood samples were collected into 3.2% trisodium citrate (Vacuette tubes, Greiner Bio-One, Alphen a/d Rijn, The Netherlands). All subjects had normal platelet counts, as measured with a Sysmex XN-9000 analyzer (Sysmex, Kobe, Japan). Where indicated, platelet concentrates from unknown healthy subjects were obtained from Sanquin (Amsterdam, the Netherlands), after written permission from Sanquin and full informed consent. A concentrate unit contained >2.5 x10^11^ platelets in 300 mL PAS-E solution and 30-35% plasma (Curvers et al., 2004). Concentrates were obtained at two days after blood drawing and were kept at room temperature by gently shaking.

### Method details

#### Platelet preparation

Platelet-rich plasma (PRP) was separated from citrated blood by centrifugation at 240 g for 15 min (Gilio et al., 2010). Alternatively, where indicated, platelet concentrates (pooled from 5 healthy donors of identical ABO and Rh(D) compatible blood types) were used from the Sanquin blood bank. Collected PRP or platelet concentrates were supplemented with 1:10 vol./vol. acid citrate dextrose (ACD; 80 mM trisodium citrate, 183 mM glucose, 52 mM citric acid), and centrifuged at 870 g for 15 min (Gilio et al., 2010). The platelet pellet was resuspended into Hepes buffer pH 6.6 (10 mM Hepes, 136 mM NaCl, 2.7 mM KCl, 2 mM MgCl_2_, 5.5 mM glucose and 0.1% bovine serum albumin). After addition of apyrase (1 U/mL) and ACD (1:15 vol./vol.), another centrifugation step was performed to obtain washed platelets (Feijge et al., 2004). Platelet pellets were resuspended into Hepes buffer pH 7.45 at desired platelet concentration (10 mM Hepes, 126 mM NaCl, 2.7 mM KCl, 2 mM MgCl_2_, 5.5 mM glucose and 0.1 % bovine serum albumin) (Gilio et al., 2010).

#### Cytosolic Ca^2+^ measurements with Fura-2

Washed platelets (200 × 10^9^/L) were loaded with Fura-2-AM (3 μM) with pluronic (0.4 μg/mL) for 40 min at room temperature. After another wash step and resuspension of the platelets at 200 × 10^9^/L, changes in [Ca^2+^]_i_ were measured in the presence of 1 mM CaCl_2_ in 96-well plates using a FlexStation 3 (Molecular Devices) at 37°C during 10 min (Jooss et al., 2019). In brief, 200 μL of Fura-2-loaded cells were stimulated by automated pipetting with 20 μL of indicated agonist solution. Mixing of agonist with cells was thus provided by high-speed injection of 10% volume of the agonist solution. Prior to default use, injection volume and speed were optimized to obtain highest platelet responses. Ratiometric changes in Fura-2 fluorescence were measured over time at dual excitation wavelengths of 340 and 380 nm, and emission wavelength of 510 nm. For nanomolar calibration of ratio values to [Ca^2+^]_i_, Fura-2-loaded platelets in separate wells were lysed by 0.1% Triton-X-100 in the presence of either 1 mM CaCl_2_ or 9 mM EGTA/Tris. The same method and machine, but at single wavelength excitation at 488 nm and no calibration, was used to measure fluorescence changes in Calcium-6 loaded platelets in 96-well plates.

#### Cytosolic Ca^2+^ measurements at UHT with Calcium-6

For UHT 384- or 1536-well plate formats, freshly obtained, washed platelets (400 × 10^9^/L) were incubated at 1:1 vol./vol. with Calcium-6 dye solution for 2 h at room temperature, according to the manufacturer's instructions. After another wash, the loaded platelets in buffer containing 1 mM CaCl_2_ were resuspended at 200-400 × 10^9^ platelets/L and distributed over 384-well or 1536-well plates. Dispersion of the platelet suspension over the 384-well plates was using a multi-pipette; dispersion over the 1536-well plates was with a Certus device (Gyger AG, Gwatt, Switzerland). In each setting, a camera-equipped FLIPR-Tetra machine (Molecular Devices) was used to measure fluorescence increases, representing rises in [Ca^2+^]_i_, in all wells simultaneously (Famili et al., 2019). In the 384-well format, 50 μL of dye-loaded platelets (200 × 10^9^/L) per well were injection pipetted with 5 μL agonist or vehicle solution. In the 1536-well format, 6 μL of dye-loaded platelets (400 × 10^9^/L) per well were injection pipetted with 2 μL of agonist or vehicle solution. As a default procedure for this machine, mixing of the platelets with agonist was achieved by high-speed injection of a relatively large volume of agonist solution. In a pilot test, injection speed and volume were optimized per well-plate format to obtain highest and most reproducible platelet responses. Using a suitable optical filter set, the increases in Calcium-6 fluorescence were continuously measured at excitation and emission wavelengths of 485 nm and 525 nm, respectively (room temperature). All conditions were repeated in duplicate to quadruplicate wells (16 wells for Z' assessment). Off-line, the time traces per well were semi-ratioed by comparison in comparison to the baseline fluorescence (F/F_o_) of the agonist medium-diluted signal (Heemskerk et al., 2001). Of note, the injection mixing in UHT assays can result in a diffusion-limited delay in response in comparison to stirred measurements, but the high curve reproducibility compensates for fastest kinetics.

#### Quantification and analysis of UHT platelet assay

Raw traces of changes in [Ca^2+^]_i_ in Calcium-6 loaded platelets were used to determine initial slope of the increase, maximal fluorescence signal increase (maximum - minimum), and area-under-the-curve (over 5–10 min). For each of these parameters, the assay's suitability was determined from the statistical effect size as Z' factor. This coefficient integrates the signal dynamic range and the test variation associated with the measured signal (Zhang et al., 1999). An assay's Z' factor is considered to be excellent with a large dynamic range and small data variability, when 1 > Z' ≥ 0.5. Assays with Z' < 0 are not useful for screening. The equation is as follows:

$$Z^{\prime }=1-\;\left(3\left(\theta p+\theta n\right)\right)/|\mu p-\mu n\;|\;$$

Herein, *θ* is the standard deviation; *μ* is the average signal value; *p* is a positive, minimum signal (resting platelets); and *n* is a negative, maximum signal (stimulated platelets).

For the modeling of [Ca^2+^]_i_, traces were separated into early and late slopes (rates of change, RoC). Traces of F/F_0_ over time were regressed to a smoothed curve (using a local weighted regression algorithm (loess, span = 0.1 and degree = 1, implemented in R version 3.2.5). Early RoC was determined as the maximum differential between 50–200 s; late RoC as the mean change over 200–450 s.

#### Use of robustness set compound library

A 263 chemical compound library for assessment of assay liabilities and suitability of UHT assays for screening of small molecules was composed by Pivot Park Screening Center (Oss, The Netherlands) (Honarnejad et al., 2021). For use of this robustness set, 4 μL of dye-loaded platelets (400 × 10^9^/L), obtained from platelet concentrates, in 1536-well format were pre-incubated by automated pipetting with 2 μL of each compound (10 μM, f.c.) or vehicle in selected wells, and after 5 min stimulated with either CRP or thrombin, as described above.

Effect percentages per compound and agonist were calculated from the mean traces of replicate wells. Three curve parameters (slope of initial increase, maximum - minimum signal, and area-under-the-curve) were always obtained, and each parameter was used to calculate a so-called Z-score. The Z-score or standard score represents the number of standard deviations above or below the mean (DeVore, 2017). Compounds were named ‘active’ (*i.e.* assay interfering) when they surpassed a Z-score threshold of > 4 or <-4, in agreement with earlier UHT protocol standards (Honarnejad et al., 2021).

#### Light transmission aggregometry and flow cytometry

For comparative measurements of platelet functions and [Ca^2+^]_i_ rises, aggregation of platelets in platelet-rich plasma or in wash platelets was determined by light transmission aggregometry. In brief, washed platelets or PRP (250 × 10^9^ platelets/L) were pre-incubated with indomethacin (10 μM) or DMSO (vehicle) for 10 min at 37°C. Maximal aggregation was induced by CRP (1 μg/mL) or thrombin (1 nM) while stirring at 1200 rpm at 37°C. Platelet aggregation was monitored using a Chronolog optical aggregometer (Havertown, PA, USA). Maximal amplitude was quantified at 10 min after the addition of the agonist. In other platelet subsamples, washed platelets (100 × 10^9^/L) were stimulated for 15 minutes with CRP (1 μg/mL), thrombin (1 nM), or left unstimulated, in the presence of 1 mM CaCl_2_. After stimulation, platelets were stained for Alexa Fluor-647 anti-CD62P mAb (2.5 μg/mL) and fluorescein isothiocyanate (FITC)-PAC1 mAb (1.25 μg/mL) to measure α-granule secretion and integrin αIIbβ3 activation, respectively. Measurements were in duplicates with an Accuri C6 flow cytometer (10,000 events) and data were analyzed with FlowJo software.

### Quantification and statistical analysis

Data were expressed as means ± SEM. GraphPad Prism 8 (San Diego, CA, USA) was used for the statistical analyses. Data normality was verified using a Shapiro-Wilk test, after which one tailed Student’s t-test or non-parametric Mann-Whitney test was used. For more than one group comparisons, a one-way repeated ANOVA test was used. Statistical significance was defined as P<0.05. Data sets were compared using Pearson or Spearman correlation analysis. The software R version 3.2.5 was used to calculate rates of changes, correlation analyses and for heatmap visualization. Statistical details per experiment are indicated in the figure legends.

## Data Availability

All data and code reported in this paper is available and will be shared by the lead contact upon request. This paper does not report original code. Any additional information required to reanalyze the data reported in this paper is available from the lead contact upon request.
