# Supplementary material for: Ultra-high-throughput Ca2+ assay in platelets to distinguish ITAM-linked and G-protein-coupled receptor activation
Source: iScience. 2021 Dec 31;25(1):103718. doi: 10.1016/j.isci.2021.103718 (PMC8762394; doi:10.1016/j.isci.2021.103718)
Supplement: Document S1. Figures S1–S6 [file mmc1.pdf]

iScience, Volume *25*

Supplemental information

Ultra-high-throughput Ca^{2+} assay in platelets to distinguish ITAM-linked and G-protein-coupled receptor activation

Delia I. Fernández, Isabella Provenzale, Hilaire Y.F. Cheung, Jan van Groningen, Bibian M.E. Tullemans, Alicia Veninga, Joanne L. Dunster, Saman Honarnejad, Helma van den Hurk, Marijke J.E. Kuijpers, and Johan W.M. Heemskerk

ISCIENCE-D-21-02226R1

Supplemental figures

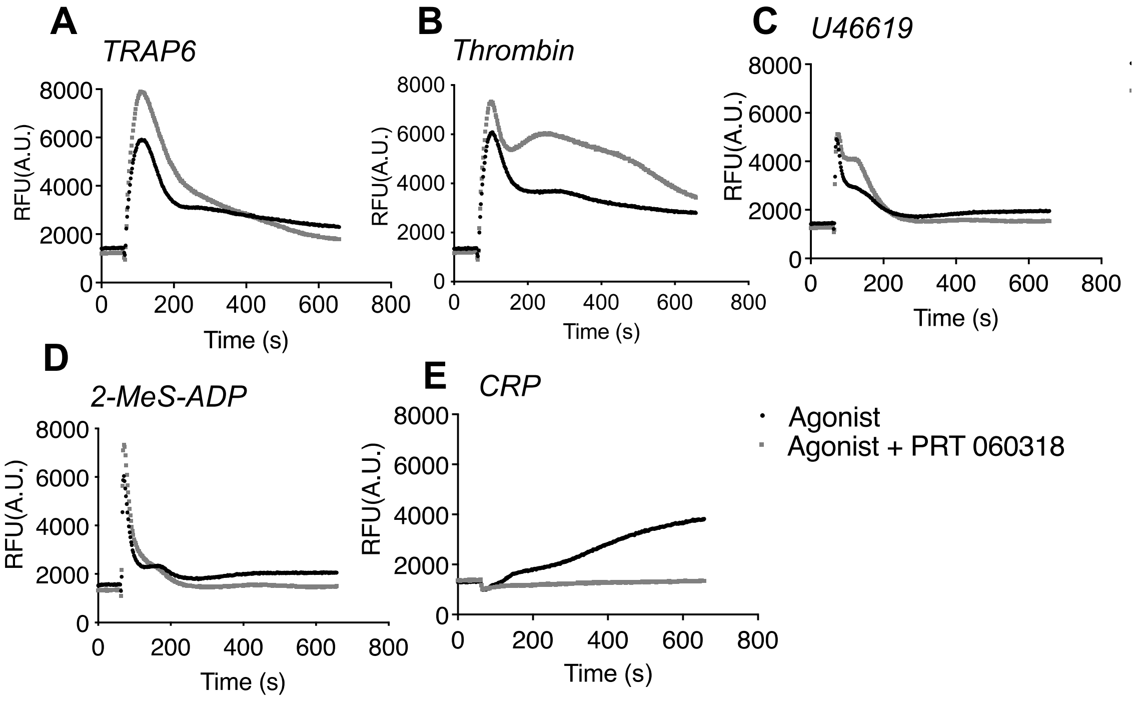

Figure S1. Differential inhibitory effect of Syk inhibitor PRT060318 on platelet $[Ca^{2+}]_i$ rises induced by ILR in comparison to GPCR agonists, related to Figure 2. Calcium-6 loaded platelets in 384-well plates were stimulated with GPCR agonists TRAP6 (10 mM), thrombin (4 nM), U46619 (10 mM) or 2-MeS-ADP (10 µM), as well as stimulated with ILR agonist CRP (10 mg/mL). The loaded platelets were pretreated for 10 min with PRT060318 (5 mM), where indicated. Changes in fluorescence per well were simultaneously recorded over time using the FLIPR-Tetra machine. Raw fluorescence traces of a representative experiment are shown.

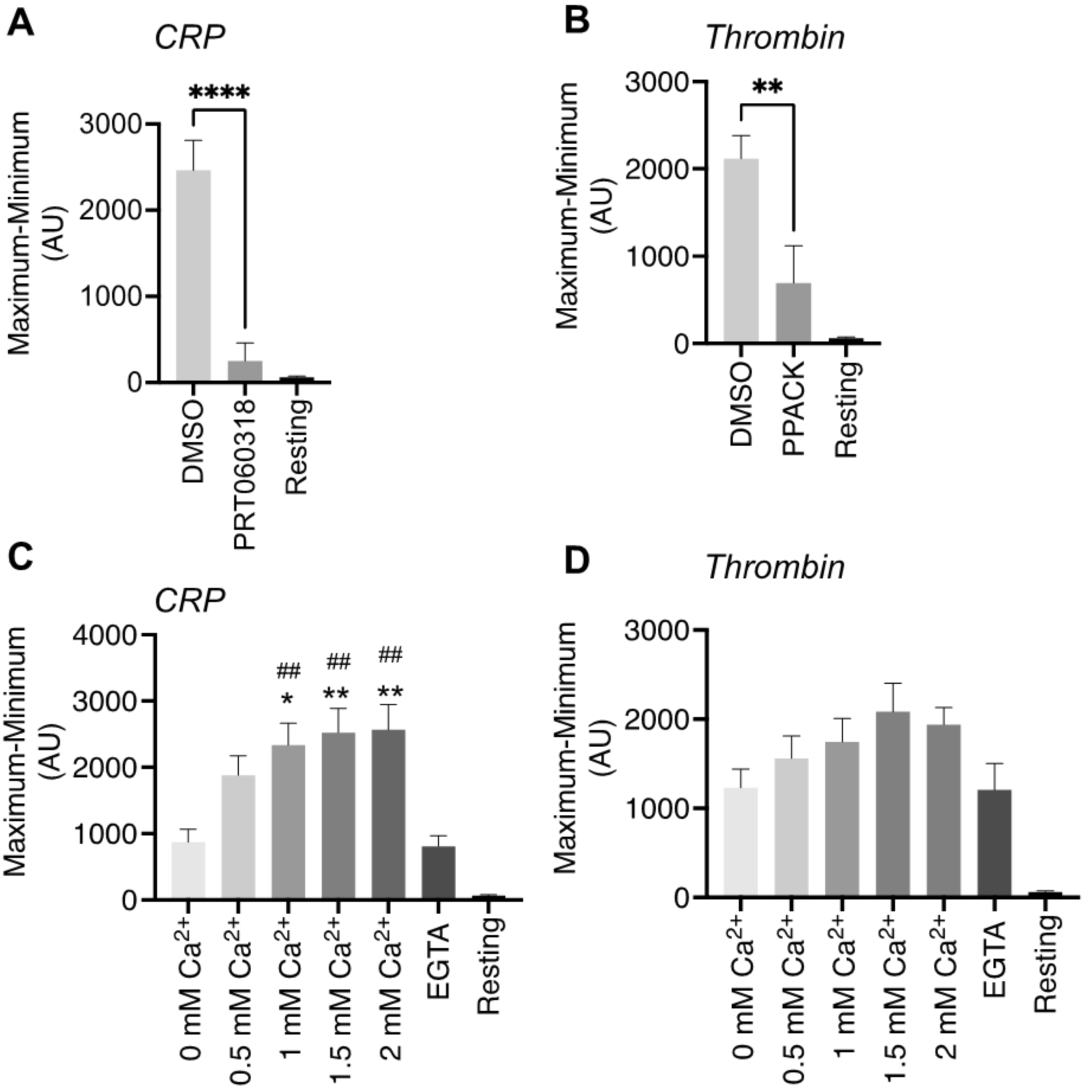

Figure S2. Agonist and $CaCl_2$-dependent characterization of platelet $[Ca^{2+}]_i$ rises, related to Figure 2. Calcium-6 loaded platelets in 96-well plates were pre-treated for 10 min with Syk tyrosine kinase inhibitor PRT060318 (10 µM), DMSO (vehicle) or thrombin inhibitor PPACK (50 nM). The cells were subsequently stimulated with CRP (10 µg/mL) or thrombin (4 nM) or left untreated (resting) (**A, B**). Means ± SEM (n = 3 donors), Student-*t*-test A-B, **P <0.01, ****P <0.0001 vs. DMSO control. (**C**, **D**) Calcium-6 loaded washed platelets were suspended in buffer medium containing 0-2 mM $CaCl_2$ or 0.1 mM EGTA, and subsequently stimulated with CRP or thrombin as above. Means ± SEM (n = 3 donors), One-way ANOVA, *P <0.05, ** P<0.01 vs. 0 mM Ca^{2+}, ## P<0.01 vs. EGTA. Shown are maximum - minimum increases in dye fluorescence over 600 s by CRP (**A**, **C**) or thrombin (**B**, **D**).

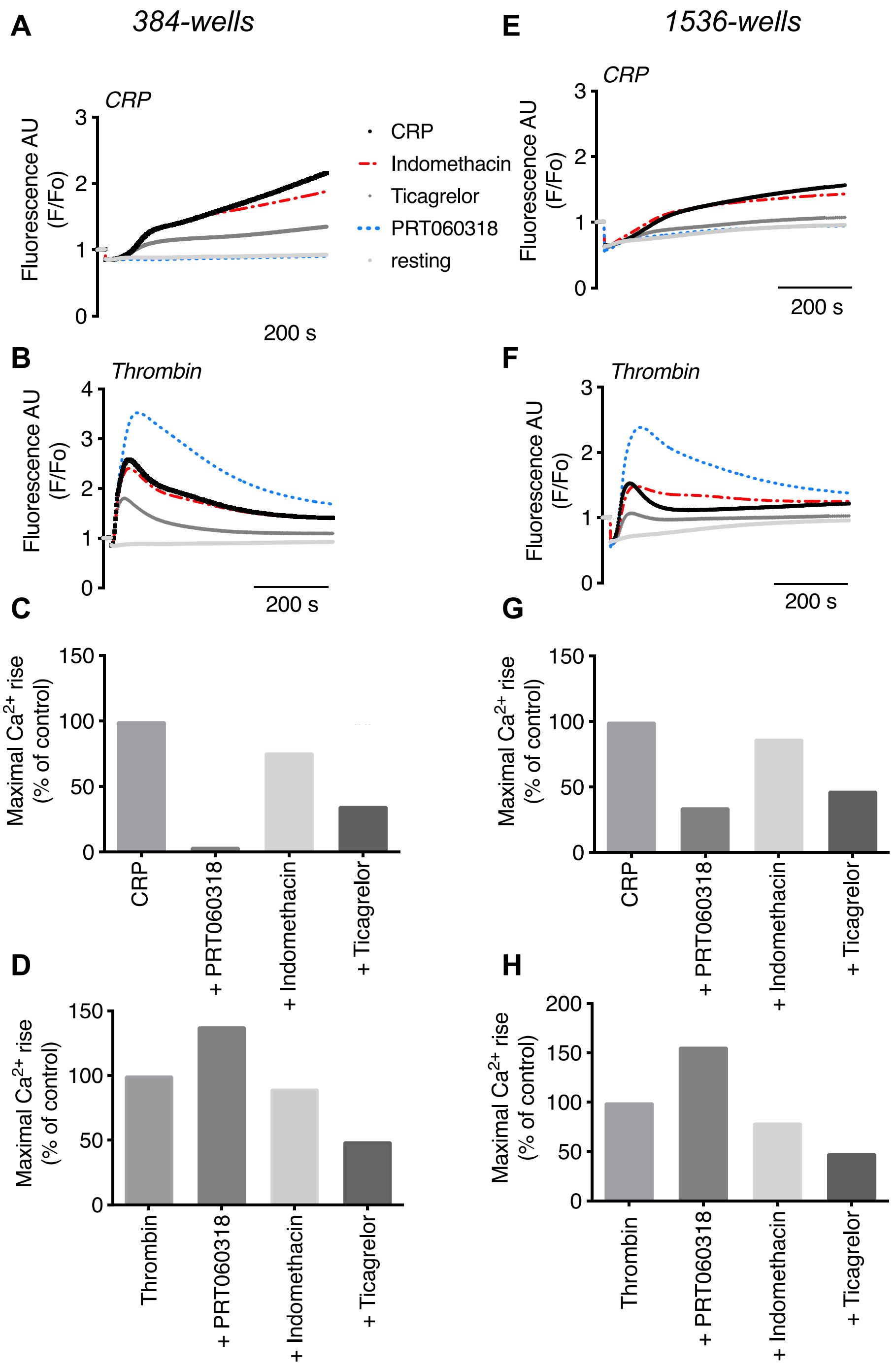

Figure S3. Single donor comparison of drug effects on agonist-induced [Ca^{2+}]$_i$ rises in 384- and 1536-well formats, related to Figure 3. Calcium-6 loaded platelets from one anonymous donor were distributed over a 384-well plate (200 x 10^9/L) and a 1536-well plate (400 x 10^9/L). The cells were pretreated for 10 min with indicated drug or vehicle (control), after which fluorescence changes per well were measured upon injection with CRP (10 µg/mL) or thrombin (4 nM) using a FLIPR-Tetra machine for 600 s, as described for Fig. 6. Time traces of changes in pseudo-ratio fluorescence (F/F_o) were then constructed per well. (**A-D**) Platelet responses in 384-wells (50 µL platelet suspension, injection of 5 µL agonist solution). (**E-H**) Platelet responses in 1536-wells (6 µL platelet suspension, injection of 2 µL agonist solution). Representative F/F_o traces of resting, CRP- or thrombin-stimulated platelets, as measured in 384-wells (**A**, **B**) or 1536-wells (**E, F**). Drugs were used as in Fig. 6 (final concentrations): PRT060318 (5 µM, blue), indomethacin (10 µM, red), ticagrelor (10 µM, grey). (**C**, **D**) Drug effects on CRP- and thrombin-induced maximum [Ca^{2+}]$_i$ rises in 384-well plate. (**G**, **H**) Drug effects on CRP- and thrombin-induced maximum [Ca^{2+}]$_i$ rises in 1536-well plate. Values expressed as percentages relative to corresponding control.

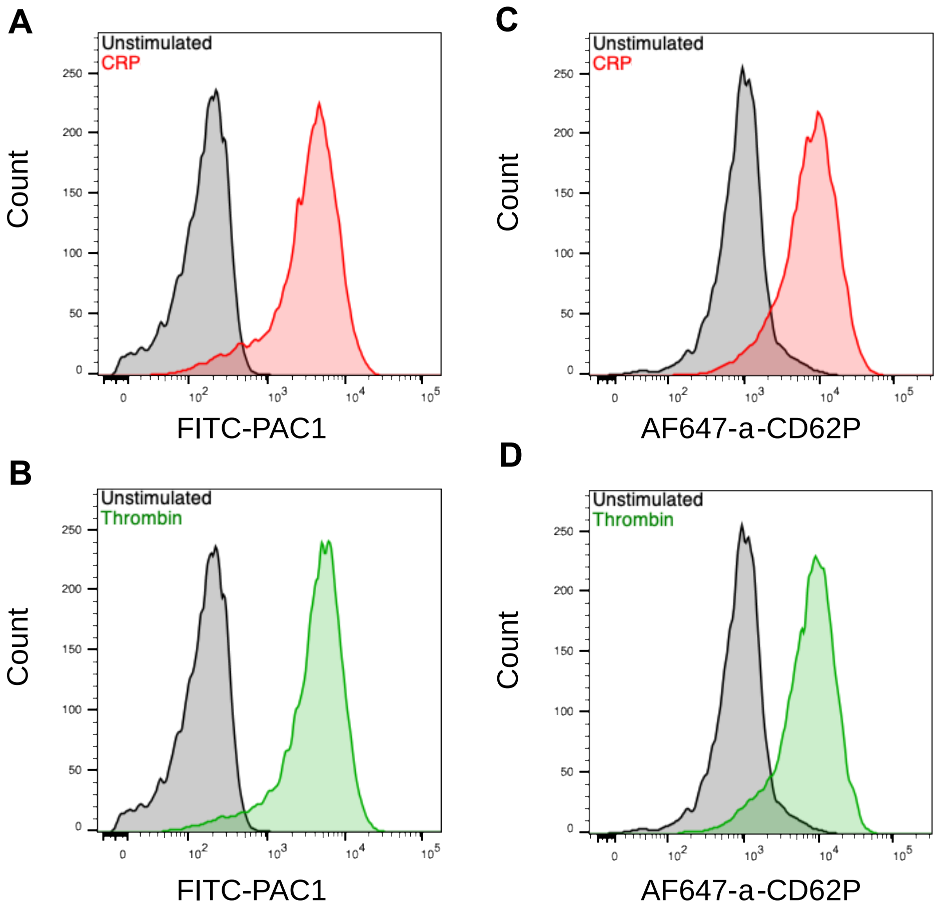

Figure S4. Agonist-induced effects on platelet activation markers by flow cytometry, related to Figure 3. Washed platelets were stimulated with CRP (1 µg/mL) or thrombin (1 nM) for 15 minutes. After stimulation, the cells were labelled FITC-PAC1 mAb (integrin αIIbβ3 activation marker) or AF647-anti-αCD62P (P-selectin exposure). Shown are representative histograms. Color code representing different samples: unstimulated platelets (grey), platelets stimulated with CRP (red), and platelets stimulated with thrombin (green). Platelets stimulated with CRP (**A**) or thrombin (**B**) and stained with FITC-PAC1mAb. Platelets stimulated with CRP (**C**) or thrombin (**D**) and stained with AF- anti-αCD62P mAb.

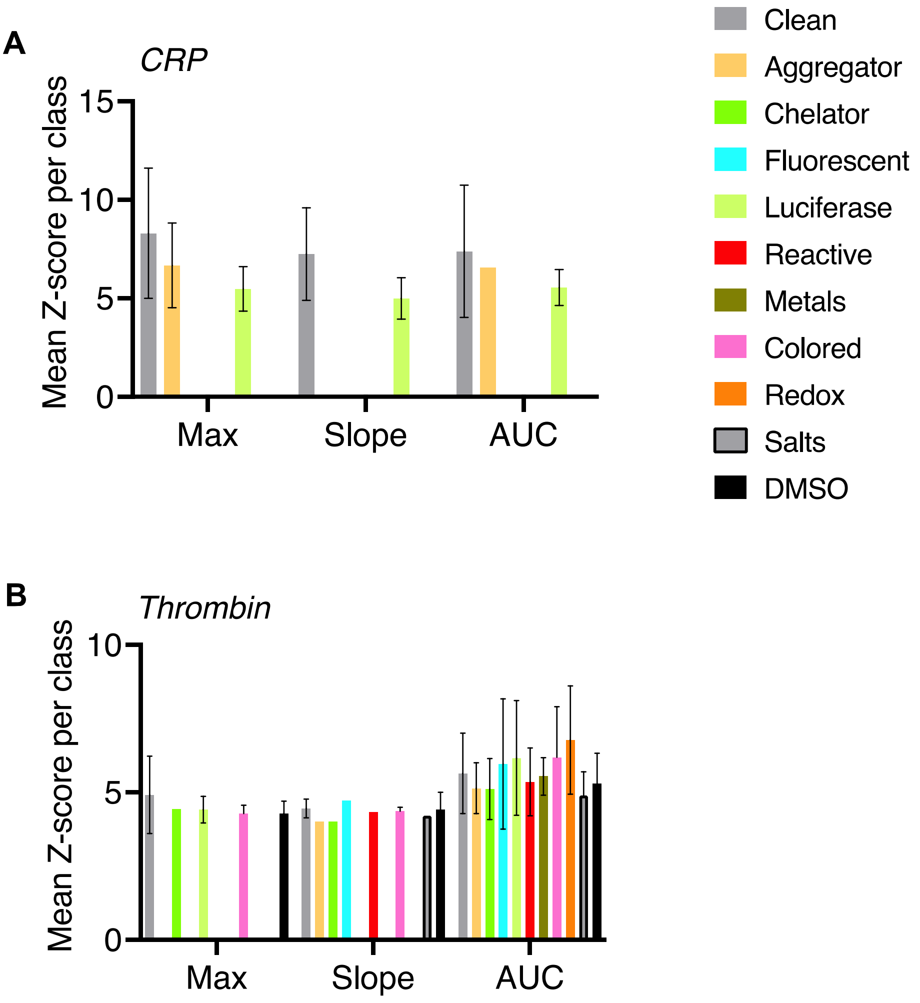

Figure S5. Interference sensitivity of UHT agonist-induced $[Ca^{2+}]_i$ assaying of Calcium-6 loaded platelets per class in UHT assay, related to Figure 5. Rises in $[Ca^{2+}]_i$ of Calcium-6 loaded platelets were measured in response to CRP (10 µg/mL), or thrombin (4 nM) in 1536-well format during 600 s. Replicate wells were preincubated with one of 263 compounds from a robustness set compound library, as in Fig. 5. Compounds were classified according to their potential assay interference: clean compounds (non-interfering); aggregator; metal ion chelator; fluorescent; luciferase quenching; chemically reactive; metal; colored or visible light absorbing; redox active; salts; and DMSO controls. Effects of active compounds (Z-score>4 or <-4) per class (see Fig. 5) are shown on Z-scores for means of $[Ca^{2+}[_i$ curve parameters, namely maximal increase (Max), curve slope, and area-under-the-curve (AUC). Data were resolved for CRP stimulation (**A**) and thrombin stimulation (**B**).

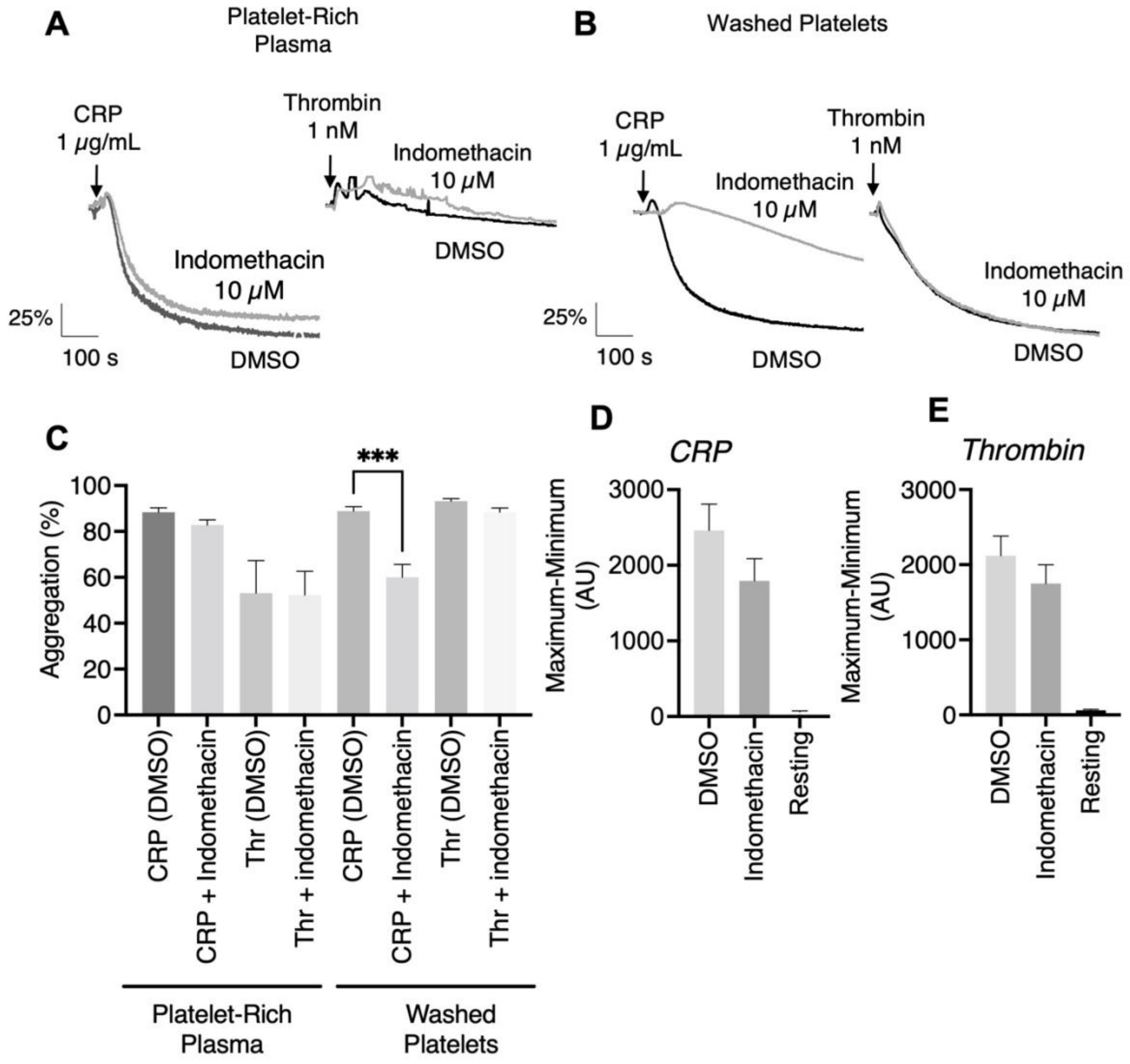

Figure S6. Comparative effects of indomethacin on agonist-induced platelet aggregation and [Ca^{2+}]$_i$ rises in Calcium-6 loaded platelets, related to Figure 6. (A-C) Platelet-rich plasma or suspended washed platelets were pre-treated with indomethacin (10 µM) or DMSO vehicle for 10 min, and then stimulated with CRP (1 µg/mL) or thrombin (1 nM). Platelet aggregation was determined by light transmission aggregometry. Shown are (**A, B**) representative aggregation traces in PRP (**A**) or washed platelets (**B**), and maximal changes in % aggregation as % transmission (**C**). (**D, E**) Calcium-6 loaded platelets from the same donors in 96-well plates were pretreated with indomethacin (10 µM) or DMSO (vehicle), and then stimulated with CRP (10 µg/mL) or thrombin (4 nM). Changes in Calcium-6 fluorescence during 600 s were recorded. Means ± SEM (n=3 donors), Student-*t*-test, *** P <0.005 vs agonist (DMSO).
